# Site-Specific Tryptophan Labels Reveal Local Microsecond–Millisecond Motions of Dihydrofolate Reductase

**DOI:** 10.3390/molecules25173819

**Published:** 2020-08-22

**Authors:** Morgan B. Vaughn, Chloe Biren, Qun Li, Ashwin Ragupathi, R. Brian Dyer

**Affiliations:** 1 Division of Natural Sciences, Oglethorpe University, Atlanta, GA 30319, USA; 2 Department of Chemistry, Emory University, Atlanta, GA 30322, USA; cebiren@aol.com (C.B.); qun.li@emory.edu (Q.L.); ashwinragu@gmail.com (A.R.)

**Keywords:** enzyme, dynamic, conformation, motion, relaxation, spectroscopy, fluorescence, tryptophan, DHFR

## Abstract

Many enzymes are known to change conformations during their catalytic cycle, but the role of these protein motions is not well understood. *Escherichia coli* dihydrofolate reductase (DHFR) is a small, flexible enzyme that is often used as a model system for understanding enzyme dynamics. Recently, native tryptophan fluorescence was used as a probe to study micro- to millisecond dynamics of DHFR. Yet, because DHFR has five native tryptophans, the origin of the observed conformational changes could not be assigned to a specific region within the enzyme. Here, we use DHFR mutants, each with a single tryptophan as a probe for temperature jump fluorescence spectroscopy, to further inform our understanding of DHFR dynamics. The equilibrium tryptophan fluorescence of the mutants shows that each tryptophan is in a different environment and that wild-type DHFR fluorescence is not a simple summation of all the individual tryptophan fluorescence signatures due to tryptophan–tryptophan interactions. Additionally, each mutant exhibits a two-phase relaxation profile corresponding to ligand association/dissociation convolved with associated conformational changes and a slow conformational change that is independent of ligand association and dissociation, similar to the wild-type enzyme. However, the relaxation rate of the slow phase depends on the location of the tryptophan within the enzyme, supporting the conclusion that the individual tryptophan fluorescence dynamics do not originate from a single collective motion, but instead report on local motions throughout the enzyme.

## 1. Introduction

Enzyme dynamics across a wide range of timescales are important for enzymatic catalysis. Conformational changes and protein motions have been implicated in every step of catalysis from crossing the barrier of the chemistry step on the femtosecond timescale to rotating whole domains on the order of milliseconds [1]. Dihydrofolate reductase (DHFR) is a classic model for studying enzyme dynamics and remains an active area of research [2,3,4,5,6,7,8,9]. DHFR is a small flexible enzyme with multiple mobile loops. In particular, the Met20 loop changes conformation throughout the catalytic cycle: it is closed in the substrate-bound complexes and occluded in the product-bound complexes [10]. Enzyme dynamics are paramount for DHFR function. It is well established that mutations that decrease loop flexibility impair DHFR enzymatic activity [11,12], but important conformational changes are not limited to the flexible loops. Molecular dynamics simulations and bioinformatics have proposed a network of correlated motions that extends throughout DHFR [13]. Experimental evidence supports that G121, F125, M42, and I14 are part of the network of correlated amino acids, demonstrating that motions near and far from the active site are important for catalysis [6]. 

**Figure 1:**
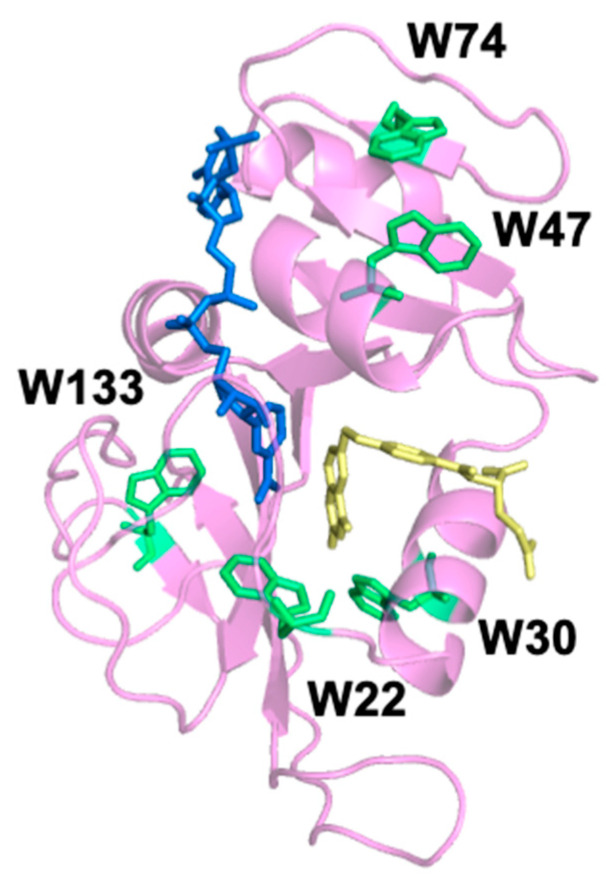
Crystal structure (Protein Data Bank: 1RX2) of wild-type E. coli dihydrofolate reductase (DHFR) (magenta) with oxidized nicotinamide adenine dinucleotide phosphate (NADP^+^) cofactor (blue) and folate (yellow). The five native tryptophan (Trp) residues are labeled and shown in green.

Recently, temperature jump (T-jump) fluorescence spectroscopy has been used to examine the dynamics of DHFR on the microsecond to millisecond timescale [14]. Laser-induced T-jump spectroscopy is unique in that it can access dynamics from tens of nanoseconds to several milliseconds. This complete time regime is difficult to access with techniques traditionally used to study enzyme dynamics such as NMR spectroscopy, hydrogen deuterium exchange (HDX), stopped-flow, and molecular dynamics simulations [15,16,17,18]. Additionally, because T-jump is a type of relaxation methodology, analyzing T-jump transients provides information about both the forward and reverse processes, which allows us to determine the relationship between relaxation events based on their concentration dependence. Previously, we measured the dynamics of wt-DHFR with T-jump spectroscopy using tryptophan (Trp) fluorescence as our probe [14]. DHFR has five native tryptophans (W22, W30, W47, W74, W133) located throughout the enzyme, as shown in Figure 1. W22 is located on the Met20 loop; W133 is located at the terminus of a beta sheet near the FG loop; W30 is near the substrate-binding site; and W47 and W74 are close together but removed from the active site. We observed relaxation rates on a microsecond timescale strongly coupled to ligand association/dissociation and a slower conformational change that was not correlated to ligand binding. Since wild-type DHFR (wt-DHFR) has multiple tryptophans, the previous study could not identify the origin of the observed slow conformational change. 

Here, we characterize the equilibrium fluorescence and T-jump dynamics of DHFR mutants, each with a single tryptophan residue (denoted as midW mutants). We found that all of the midW mutants exhibited biphasic relaxation rates similar to wt-DHFR with a concentration-dependent fast relaxation rate and a concentration-independent slow relaxation rate. Notably, the slow relaxation rate of the midW mutants differs based on the location of the Trp probe within the enzyme and is generally faster than the slow event observed previously in wt-DHFR. This evidence supports the conclusion that the observed relaxation rate in wt-DHFR likely arises from conformational changes that modulate the Trp–Trp interactions and that individual tryptophan residues report on local motions.

## 2. Results and Discussion

### 2.1. Protein Characterization 

To verify that the Trp to phenylalanine (Phe) mutations did not adversely affect the stability and function of midW mutant DHFR enzymes, the melting temperature and activity of the enzymes were examined. The effects of the Trp to Phe mutations on enzyme catalysis were determined by comparing wt-DHFR and midW mutant activity by monitoring nicotinamide adenine dinucleotide phosphate (NADPH) absorbance at 340 nm. As shown in Table S1**,** the midW22 mutant retained 90% activity compared to the wild-type enzyme. The high activity of midW22 compared to wt-DHFR demonstrates that W30, W47, W74, and W133 have little importance in the catalytic activity of DHFR, since the midW22 mutant has all of the Trp residues, except W22 is replaced with Phe. The other four midW mutants have reduced activity (20–33%), which can be explained by disrupting the hydrogen bond network between W22, D27, T113, and the substrate DHF [19].

**Figure 2:**
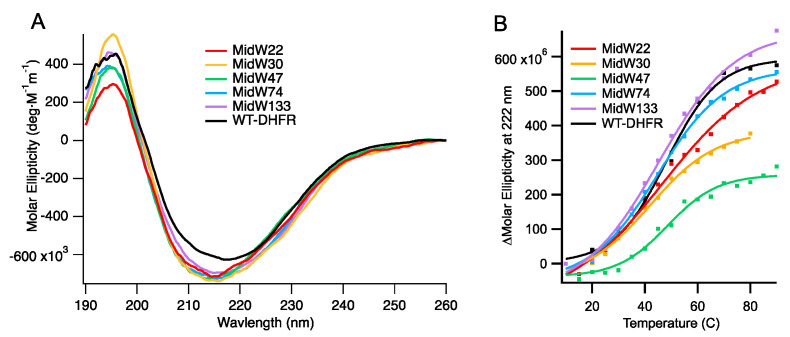
(**A**) Circular dichroism (CD) spectra of wild-type DHFR (wt-DHFR) and the five midW mutants. Trp side chains can impact the CD spectra. Wt-DHFR has five trp residues, whereas the midW mutants have only one trp each. Despite the lower molar ellipticity of the mutants compared to the wild type, the CD spectra show that the mutants are folded enzymes with both alpha helices and beta sheets. (**B**) Thermal melts of wt-DHFR and the midW mutants. midW: DHFR mutants, each with a single tryptophan residue.

Circular dichroism (CD) spectroscopy was used to examine the fold and the stability of the midW mutants. Figure 2A shows the CD spectra. Notably, the molar ellipticity of the midW mutants is lower compared to the wild-type enzyme. In the midW mutants, all but one of the five native Trp residues have been mutated to Phe. Previously, single Trp to Phe, valine, or leucine mutations have been shown to impact the CD spectra from 190 to 250 nm [20]. The mutations caused a loss of ellipticity, gain of ellipticity, or a mixture of the two in different regions of the CD spectra depending on the location of the Trp mutation and which amino acid was chosen to replace the Trp residue. In particular, exciton coupling between W47 and W74 has a large impact on the CD spectra of DHFR. In each of the midW mutants, W47, W74, or both have been removed, making a direct comparison to the wt-DHFR spectra difficult to interpret. However, the CD spectra does show that the midW mutants have a minimum band in molar ellipticity around 215 nm and a maximum around 195 nm, which is characteristic for folded proteins with both alpha helices and beta sheets. The melting temperatures of the midW mutants were determined by monitoring the loss of ellipticity at 222 nm with increasing temperatures via CD spectroscopy (Figure 2B). We determined the melting temperature of wt-DHFR to be 48 ± 1 °C, which is similar to the previously determined melting temperature of 49.3 °C [21]. The melting temperatures of the midW mutants range from 42 to 49 °C (Table 1). The melting temperature is an important consideration, since the proteins are transiently heated to 36 °C during the T-jump experiments. For midW22, midW47, midW74, and midW133, the final temperature is 9 °C or more below the melting temperature, so unfolding is not likely to play a large role. MidW30 has a lower melting temperature of 42 °C, so unfolding effects may be relevant to the observed dynamics. 

### 2.2. Equilibrium Fluorescence

**Figure 3:**
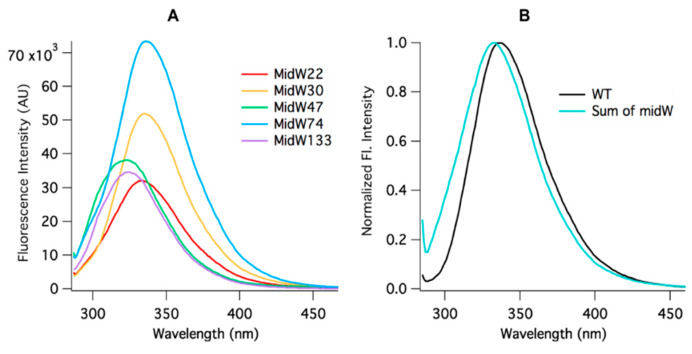
(**A**) Trp fluorescence of the apoenzymes of the five midW mutants (3 μM). Differences in fluorescence intensity and shift indicate that each tryptophan is in a unique environment. (**B**) Wt-DHFR Trp fluorescence spectrum and the summation of the midW mutants′ fluorescence spectra. Wt-DHFR has a much greater fluorescence intensity than the individual midW mutants combined. The intensities have been normalized here to better demonstrate the blue shift of the summed spectra compared to the wild-type spectrum. This data show that the wt-DHFR Trp fluorescence is not a simple linear combination of each individual tryptophan’s fluorescence.

The tryptophan fluorescence spectra of all five midW mutants were measured for the apoenzyme and three different enzyme·ligand complexes: E·Folate, E·NADP^+^, and E·NADP^+^·Folate. Each of these enzyme–ligand complexes are model systems for the different enzyme states in the catalytic cycle: the binary product complex, the holoenzyme, and the Michaelis complex, respectively. The fluorescence intensity of each midW mutant was much less than the wild-type enzyme (Figure S2), as expected, since the midW mutants each contain only one tryptophan, whereas wt-DHFR contains 5 tryptophans. As shown in Figure 3A, the intensity of the Trp fluorescence of each midW mutant differs, with midW74 exhibiting the greatest fluorescence intensity, followed by midW30, midW47, midW133, and midW22 with the lowest fluorescence intensity. Additionally, the shift in the Trp fluorescence maximum between midW mutants shows that the Trp in each mutant is in a different environment. Typically, a blue shift indicates a more hydrophobic environment and tends to be concomitant with an increase in fluorescence intensity. A red shift indicates a more hydrophilic environment, which usually corresponds with a decrease in fluorescence intensity, due to quenching by water. Interestingly, the Trp fluorescence of the midW mutants does not necessarily follow these trends. For example, midW47 and midW133 are blue shifted compared to midW74 and midW30, yet midW47 and midW133 display lower fluorescence intensities. This indicates that there must be some quenching interactions with W47 and W133 in the apoenzyme. Additionally, there are likely quenching interactions at position 22, given that midW22 exhibits a similar shift compared to midW74 and midW30. The similar shift suggests a similar environment; however, midW22 Trp fluorescence is drastically reduced.

Figure 3B shows the comparison of the wt-DHFR Trp fluorescence spectrum to the sum of the Trp fluorescence spectra of the midW mutants. The intensities have been normalized to 1 to better demonstrate the difference in lambda max between the two spectra. The difference in the spectra shows that the wt-DHFR Trp fluorescence is not a simple linear combination of the fluorescence of the individual tryptophans. This has been observed previously, albeit in a less direct manner by Ohmae et al., where a single tryptophan was mutated to various hydrophobic residues [20]. They observed that the Trp fluorescence decreased in varying amounts depending on which tryptophan was mutated. In this way, they came to the same conclusion as stated above. Furthermore, W47 and W74 are known to form an excimer pair [20]. Typically, the emission of the excimer is red shifted compared to the emission of the fluorescent monomers, which may contribute to the observed red shift of the wild-type DHFR fluorescence compared to the sum of the fluorescence of the individual midW mutants.

**Figure 4:**
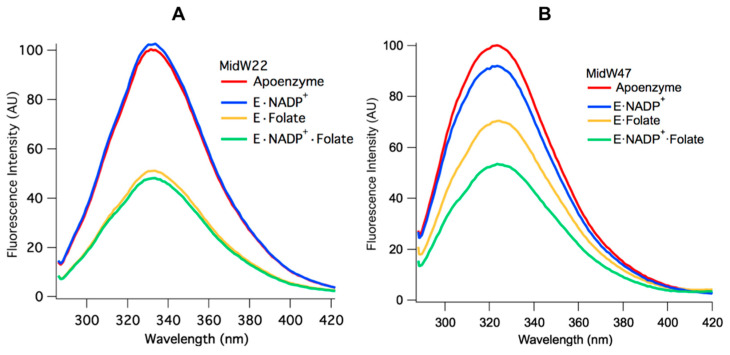
Equilibrium Trp fluorescence of midW22 (**A**) and midW47 (**B**) and their ligand complexes (3 μM enzyme with 6 μM folate and/or 6 μM NADP^+^). The Trp fluorescence of midW22 appears to be primarily modulated by the presence of folate, whereas the Trp fluorescence of midW47 changes with each ligand complex.

Two patterns emerge when the Trp fluorescence of the midW mutant ligand complexes are compared. Figure 4A shows the midW22 mutant complexes, which exhibit the same trend as all the other mutants except for midW47, which is shown in panel B. Table 2 shows the integrated fluorescence intensity of each complex as a percentage of the apoenzyme fluorescence intensity. MidW22 and the other three midW mutants (midW30, midW74, and midW133) show very little change upon binding NADP^+^ compared to the apoenzyme and show a significant change compared to apoenzyme for the binary complex with folate and the tertiary complex. Importantly, there is only a small difference in the Trp fluorescence intensity between the E·Folate and E·NADP^+^·Folate complexes, which exist in the occluded and closed conformations, respectively. This indicates that W22, W30, W74, and W133 are not particularly sensitive to the closed versus occluded conformational states of the Met20 loop and are instead primarily sensitive to the presence of folate. Conversely, the Trp fluorescence of midW47 (Figure 4B) decreases by different amounts based on which ligands are bound. There is a small decrease upon binding NADP^+^, a larger decrease upon binding folate, and a further decrease in the tertiary complex. However, the combined decrease in fluorescence intensity of individually binding folate and NADP^+^ is less than the total decrease observed for the tertiary complex. This means that the decrease in fluorescence intensity observed in the tertiary complex is not simply due to quenching of the Trp fluorescence from the presence of ligands; there is also a contribution from the conformation of the Met20 loop. Thus, midW47 is more sensitive to changes in the enzyme conformation compared to the other midW mutants.

**Figure 5:**
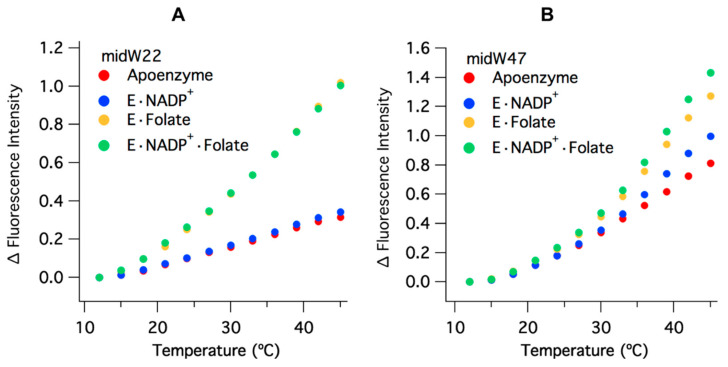
Change in fluorescence intensity versus temperature, corrected for the intrinsic temperature dependence of Trp fluorescence, of (**A**) midW22 and (**B**) midW47 (3 μM enzyme with 6 μM folate and/or 6 μM NADP^+^). All complexes demonstrate an increase in fluorescence with increasing temperature. The change in intensity for the binary complex with NADP^+^ and apoenzyme compared to the two folate bound complexes is similar for midW22, which is expected based on the similarities in the spectra of apoenzyme/E·NADP^+^ and E·Folate/E·NADP^+^·Folate. Conversely, each midW47 complex has a slightly different temperature dependence, reflecting the difference in Trp fluorescence intensity for the different ligand bound states.

The temperature-dependent Trp fluorescence of the midW mutants is shown in Figure 5. The values have been corrected for the intrinsic temperature dependence of Trp fluorescence, which decreases with increasing temperature (Figure S3). This is opposite of the trend observed for the enzyme complexes. All of the midW mutants follow the same general trend as the wild-type enzyme: an increase in fluorescence intensity with increasing temperature, due primarily to ligand dissociation and accompanying loop conformation changes. Each of the midW mutants follow the trend as expected based on their change in Trp fluorescence with different ligands bound. An additional contribution to the increased Trp fluorescence is likely caused by loosening of the enzyme structure at elevated temperatures, which in turn reduces quenching interactions within the protein. This explains the increase in Trp fluorescence for the apoenzyme where no ligands are present to dissociate. Figure 5 shows the change in fluorescence intensity for midW22 and midW47 as representative data. 

### 2.3. Temperature Jump Enzyme Dynamics

**Figure 6:**
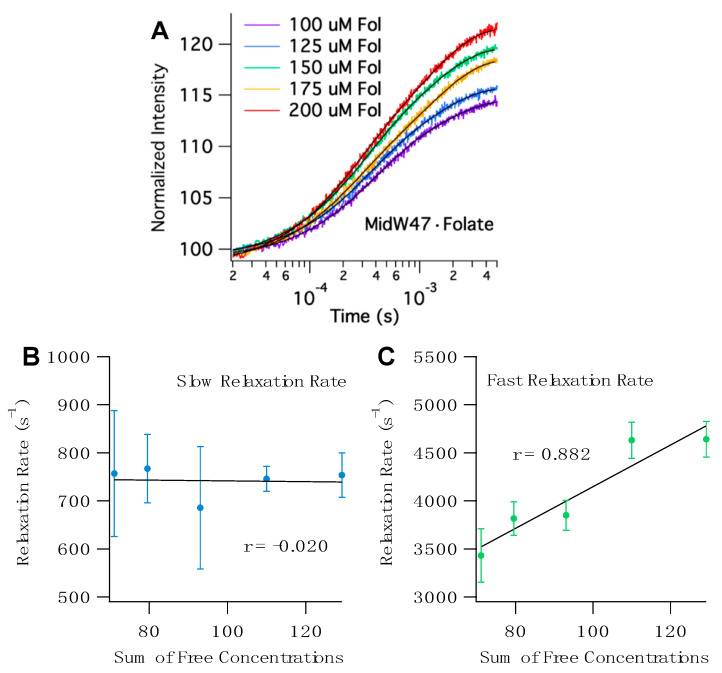
(**A**) Representative temperature jump (T-jump) Trp fluorescence transients of midW47·Folate with varying concentrations of folate. The black traces are double exponential fits. (**B**) Correlation plot for the slow relaxation rates of midW47·Folate versus the sum of the concentration of free enzyme and free ligand. The slow relaxation rate is concentration independent, as shown by the low r value. (**C**) Correlation plot for the fast relaxation rates of midW47·Folate versus the sum of free concentrations. The r value close to one confirms that the fast relaxation rate is strongly concentration dependent.

The T-jump transients for all five midW mutants with folate share a few key characteristics. The transients fit to double exponentials with two distinct phases (Tables S3 and S4). To determine the origin of the observed relaxation rates, we can examine the correlation between the sum of the concentrations of the free enzyme and free ligand. Since T-jump spectroscopy is a relaxation method that provides information about both the forward and reverse reactions, if the observed rate is concentration dependent, then there must be a bimolecular step—in this case, ligand binding. Figure 6 shows representative T-jump transients of midW47 and the concentration-dependent plots. The sum of free concentrations at 36 °C, the temperature after the laser pulse, was calculated using the K_d_ and ∆H of folate binding determined by isothermal titration calorimetry (ITC). The ITC results are summarized in Table S2. The concentration dependence of the fast and slow relaxation rates for the midW mutants are shown in Figure 6 and Figure S4. The fast relaxation rate of the midW mutants are positively correlated with the sum of the concentrations of the free enzyme and free ligand. The strength of the correlation can be quantitatively described by the linear correlation coefficient, r. The r values range from 0.867 to 0.954 for midW30, midW47, midW74, and midW133. The threshold for significance at a 99% confidence level is 0.505 (DF = 23). [22] Thus, all of the midW mutants except for midW22 are significantly correlated with concentration and can be qualitatively described as strongly concentration dependent. MidW22 exhibits a weak concentration dependence with an r value of 0.366, which is higher than the cutoff for significance at the 90% confidence level (0.337, degrees of freedom (DF) = 23). The concentration dependence of the fast relaxation rate indicates that the fast relaxation phase is convolved with ligand binding/unbinding. Conversely, the correlation coefficients of the slow relaxation rate vary from −0.659 to 0.124. A negative correlation with regard to concentration is nonsensical. Therefore, we can conclude that the slow relaxation rate of midW22, midW30, midW47, and midW133 is not concentration dependent. MidW74 has an r value of 0.124, which is a very low correlation coefficient. Even at an 80% confidence level, the midW74 correlation coefficient is lower than the threshold value (0.265, DF = 23), indicating that there is no significant correlation between the slow relaxation rate and the sum of the free concentrations. The concentration dependence of the midW mutant relaxation rates follows the same trend as the wild-type relaxation profile. A fast concentration-dependent relaxation rate and a slow concentration-independent relaxation rate were also observed for wt-DHFR [14].

Assuming a simple two-state model where a ligand binds to an enzyme to form an enzyme– ligand complex, a linear fit of the fast relaxation rate as a function of the sum of free concentrations (Figure 6C and Figure S4B,D,F,H) provides additional information. The slope of the line is equivalent to k_on_ and the y-intercept is equivalent to k_off_ [23]. The k_on_ and k_off_ values for wt-DHFR and the midW mutants are reported in Table 3. The k_on_ and k_off_ are modulated in the midW mutants compared to wt-DHFR. While k_on_ is similar to what has been reported in the literature for the DHFR–folate complex (57 ± 5 μM^−1^s^−1^) [24], the value of k_off_ is two orders of magnitude larger (35 ± 12 s^−1^) [24]. Furthermore, comparing the K_d_ values determined by ITC experiments and the K_d_ values calculated from k_off_ and k_on_ reveals a discrepancy. The K_d_ values calculated from the T-jump experiments are approximately an order of magnitude larger than the K_d_ values determined from ITC. Taken together, these results indicate that the observed fast relaxation rate is not only due to ligand association/dissociation but is also convolved with associated conformation changes, such as the Met20 or FG loop dynamics. Loop motions within DHFR are critical for enzyme activity. In particular, two mutations have been studied that impact loop flexibility and consequently impact enzyme catalysis. When N23 at the end of the Met20 loop is replaced with a double proline sequence, millisecond fluctuations of the Met20 loop are abrogated [12]. When G121 on the FG loop is replaced with a valine, the pico-nanosecond and micro-millisecond motions of both the FG and Met20 loops are decreased [11]. Both of these studies used NMR relaxation experiments and thus were limited to time regimes accessible by NMR. Since T-jump spectroscopy is capable of measuring dynamics on the nanosecond to millisecond timescale, future studies with the N23PP or G121V mutants could use T-jump spectroscopy to fill in the time gap.

Although the T-jump transients of all five mutants show similar behavior with respect to concentration dependence, the relaxation rate of the slow phase varies based on the position of the Trp. The average relaxation rates of the slow phase are reported in Table 4. Since the slow relaxation rate is concentration independent, the average rate is the combined average of the five replicates at the five ligand concentrations (n = 25). W47 and W74 are spatially close together; they are located at the top of DHFR when orientated as shown in Figure 1. MidW47 and midW74 have close average slow relaxation rates of approximately 730 s^−1^. The slow phase relaxation rates for MidW22 and midW30 are substantially faster, in excess of 1000 s^−1^ and are within one standard deviation of one another. Similarly, W22 and W30 are close together spatially; they are located near the substrate binding site. Lastly, W133 is located on a beta sheet near the distal FG loop, and midW133 has the slowest average slow phase relaxation rate of 370 s^−1^. To compare, the binary complex of wt-DHFR with folate has a slow concentration-independent relaxation rate of 400 s^−1^. This is slower than all but one of the concentration-independent rates observed in the midW mutants. A linear combination of the relaxation rates of midW mutants would not result in the relaxation rate observed in the wild-type enzyme unless the wt-DHFR transients were almost completely dominated by the W133 signal with very little contribution from the other four Trp residues. Our equilibrium fluorescence results show that midW133 has a low fluorescence intensity compared to the other midW mutants. Additionally, Ohmae et al. showed that when W133 is replaced with phenylalanine, the tryptophan fluorescence of DHFR is reduced by less than 15% [20]. Therefore, it is unlikely that W133 is dominating the fluorescence signal in wt-DHFR. Instead, Trp–Trp interactions as well as the local environment of the Trp residues contribute to the wt-DHFR transients, similar to what we observed with the equilibrium fluorescence. Thus, the motions reported in the multi-Trp system are fundamentally different than the motions reported in the single Trp systems. We conclude that the wt-DHFR signal is dominated by Trp coupling effects due to relative changes in the distance and orientation of the Trp residues, which means that the wt-DHFR transients are reporting on global motions throughout the enzyme. Conversely, the signals from the midW mutants report on local motions that affect the environment of the single tryptophan in the enzyme, as evidenced by the dependence of the slow relaxation rate on the Trp location.

By comparing the relaxation rates of the midW mutants, we can gain information about the environments of the individual Trp residues. Of particular interest are midW22 and midW30 because they both exhibit faster relaxation rates compared to the other midW mutants. The faster relaxation rate indicates that W22 and W30 are sensitive to conformational changes of a local flexible structure. MidW30 has a lower melting temperature compared to wt-DHFR and the other midW mutants. During the T-jump experiments, samples are transiently heated to 36 °C, which is 6 °C lower than the melting temperature of midW30. It is possible that unfolding events may be playing a role. Another possibility is that W22 and W30 could be reporting on the same motion. W30 is located near the active site, and W22 is located at the end of the Met20 loop, which is known to change conformations during the catalytic cycle: opening and closing over the active site as well as protruding into the active site. The conformation of the Met20 loop can be modulated by ligand binding. In the binary complex with folate, the Met20 loop is in the occluded conformation, and in the apoenzyme, the Met20 loop is in the open conformation. Since W22 and W30 are on or near the Met20 loop, it is reasonable that they could be sensitive to the Met20 loop motions. The slow relaxation rates of midW22 and midW30 are approaching that of the fast concentration-dependent relaxation rate. The strong concentration dependence of the faster relaxation rate indicates that it is strongly coupled to ligand association/dissociation. The slow relaxation rate for midW22 and midW30 is not concentration dependent, which means that the slow relaxation rate is not coupled to ligand binding. This suggests that the observed slow relaxation rate is not the open to occluded transition. However, the slow relaxation event sensed by W22 and W30 is still likely related to Met20 loop fluctuations due to the location of the Trp probes and the observed timescale. Loop motions typically occur in tens of nanoseconds to hundreds of microseconds, [1,25] and the slow relaxation events of midW22 and midW30 occur at approximately 300–500 microseconds. This is in contrast to what is observed with wt-DHFR and the other midW mutants where the slow relaxation events occur on the order of milliseconds.

## 3. Materials and Methods 

### 3.1. Mutant DHFR Plasmid Generation, Protein Expression, and Purification

C-terminal six histidine-tagged DHFR midW mutant plasmids were generated via the Custom Cloning Division within the Emory Integrated Genomics Core. In each midW mutant, all but one of the native tryptophans were mutated to phenylalanine (Phe). The prefix “mid” is old English for with; thus, midW22 is the DHFR mutant containing W22 with all other tryptophans mutated to phenylalanine. The midW mutants were expressed and purified as described previously for wild-type DHFR [14]. Briefly, BL21 (DE3) *E. coli* competent cells were transformed with the midW mutant plasmids, grown with 100 μg/mL ampicillin, induced with 1 mM isopropyl β-d-thiogalactopyranoside (IPTG) overnight, and harvested by centrifugation. The pelleted cells were lysed by sonication, and the insoluble debris was removed by centrifugation. The supernatant was purified by loading on a HisPrep affinity column and washed to remove protein contaminants as well as endogenous NADPH. Finally, the midW protein was eluted with 500 mM imidazole, buffer exchanged, concentrated, and stored at −80 °C. Protein concentration was determined by absorbance at 280 nm with a predicted molar extinction coefficient of 10,810 M^−1^ cm^−1^ [26]. 

### 3.2. Relative Activity Assay

The relative activities of the midW mutants compared to wild-type DHFR were measured as previously described [27]. Briefly, 20 μL of 2.5 mM DHF were added to 980 μL of pre-equilibrated 10 nM DHFR and 51 μM NADPH in phosphate buffer (50 mM sodium phosphate, 100 mM NaCl, 5 mM 2-mercaptoethanol, pH 7). The rate of reaction was measured by monitoring the disappearance of the 340 nm absorbance band as NADPH was oxidized. The initial rates are reported in Table S1 as a percentage of the initial rate of wild-type DHFR.

### 3.3. CD Spectroscopy and Thermal Melts

CD spectroscopy measurements were taken on a JASCO J-810 spectropolarimeter equipped with a PFD-425S Jasco temperature controller (Jasco, Inc., Easton, MD, USA) in a quartz cuvette with a 1 mm path length. Samples of wt-DHFR and midW mutants (15–30 μM) were prepared in 10 mM phosphate buffer, pH 7. The melting temperatures of the midW mutants were determined by monitoring the loss of ellipticity at 222 nm with increasing temperature. Measurements were taken in increments of 5 °C from 10 to 90 °C. To calculate the melting temperatures, the change in ellipticity at 222 nm versus temperature was fit to a sigmoid.

### 3.4. Equilibrium Fluorescence

Equilibrium fluorescence measurements of wt-DHFR apoenzyme and the midW mutants with their corresponding complexes (apoenzyme, E·Folate, E·NADP^+^, and E·NADP^+^·Folate) were taken on a Horiba (Kyoto, Japan) Dual-Fl spectrofluorometer. Spectra were obtained by exciting with 280 nm light, corresponding to tryptophan absorbance. Due to the saturation limits of the fluorometer detector, wt-DHFR spectra were collected with an integration time of 0.5 s. The spectra of the midW mutants were collected with an integration time of 1.5 s. Each sample contained 3 μM of enzyme in phosphate buffer (50 mM sodium phosphate, 100 mM NaCl, pH 7). Samples included 6 μM of the appropriate ligands for the two binary complexes. For the tertiary complex, both 6 μM folate and 6 μM NADP^+^ were present. Spectra were collected in triplicate and were averaged together. To compare changes in fluorescence intensities between complexes, the entirety of the Trp fluorescence peaks were integrated. The integrated intensities of the enzyme–ligand complexes of each mutant are reported as a percentage of the corresponding apoenzyme fluorescence intensity. Additionally, temperature-dependent fluorescence spectra were collected from 12 to 45 °C in increments of 3 °C. The Trp fluorescence was integrated between 327 and 353 nm, which corresponds to the bandpass filter used in the temperature jump experiments. The resulting intensities were normalized to 1 with respect to the lowest temperature measured, and the intrinsic temperature dependence of tryptophan was subtracted as described in the supplemental information. These results are presented as change in fluorescence intensity.

### 3.5. Temperature Jump Fluorescence Spectroscopy

Temperature jump fluorescence transients were collected and analyzed as described previously [14,28]. Briefly, the temperature jump was induced via a 2.09 μm, approximately 10 ns laser pulse at 12.5 Hz. The changes in Trp fluorescence were probed by exciting the sample with 280 nm light, focusing the emission through a bandpass filter (327–353 nm), and measuring the intensity with a photomultiplier tube. The temperature jumps reported here are from 29 to 36 °C; this range is below the melting temperatures of the midW mutants, which range from 42 to 49 °C (Table 1). The phosphate buffer that was used for the equilibrium fluorescence experiments was D_2_O exchanged for T-jump use. For each midW mutant, five samples were prepared containing 100 μM enzyme with varying concentrations of folate, up to 200 μM. The T-jump transience was collected in replicates of five. The intrinsic temperature-dependence of tryptophan was subtracted from each transient using approximately 200 μM free tryptophan as a reference. Then, the corrected fluorescence transients were normalized to 100 by dividing the entire transient by its initial fluorescence intensity, effectively reporting a percent change in fluorescence intensity over time. Finally, each transient was fit to a double exponential. 

## 4. Conclusions

In this study, we characterized the equilibrium Trp fluorescence and T-jump dynamics of five DHFR mutants, each with a single tryptophan residue. The equilibrium fluorescence confirms that each tryptophan residue is in a unique environment and that there are local quenching interactions. The wt-DHFR Trp fluorescence is not a simple combination of the individual tryptophans′ fluorescence due to Trp–Trp interactions such as excimer formation with W47 and W74. The T-jump dynamics demonstrate that all of the tryptophan residues are sensitive to ligand binding and dissociation in combination with associated conformational changes. Furthermore, the flexibility of DHFR is highlighted by the presence of slow microsecond–millisecond conformational changes throughout the enzyme that are not coupled to ligand association/dissociation processes relating to progress along the catalytic cycle. These fluctuations do not originate from a single collective motion, but instead arise from local motions with slightly different relaxation rates. Some of the relaxation rates are quite slow and likely do not impact catalysis. However, some relaxation rates, such as those observed in midW22 and midW30, are fast compared to the steady-state turnover rate of DHFR, and thus, they could be involved in conformational sampling and the search for reactive conformations.

More generally, this study explores the utility of fluorescence spectroscopy applied to multi and single Trp systems. Trp fluorescence is often used as a probe for protein and enzyme studies. Trp is a naturally occurring amino acid, so endogenous Trp residues can be used as a probe or Trp can be easily incorporated into the protein of interest. Trp fluorescence is an ideal reporter because it is sensitive to the environment of the Trp. However, interpreting changes in Trp fluorescence can be challenging, because Trp is sensitive to local changes in its environment as well as various quenching interactions, Trp–Trp interactions, and Förster resonance energy transfer (FRET) to other fluorophores. Here, we demonstrated that multi-Trp systems can be used to follow global conformational changes as a result of Trp–Trp interactions. Furthermore, we showed that working with the corresponding single Trp mutants not only provides insight about the local conformational changes and environments of the individual tryptophans but also provides the necessary information to confirm that the multi-Trp system was reporting on global conformational changes. This type of coupled multi- and single fluorophore approach is broadly applicable and could be used to study the dynamics of other protein and enzyme systems.

## Figures and Tables

**Table 1:** Melting temperatures of wild-type (WT) and midW mutants as determined by loss of ellipticity at 222 nm.

Enzyme	midW22	midW30	midW47	midW74	midW133	WT
Melting Temperature (°C)	46 ± 2	42 ± 1	49 ± 2	45 ± 2	45 ± 2	48 ± 1

**Table 2:** Integrated Trp fluorescence intensity of the enzyme–ligand complexes as a percentage of the integrated Trp fluorescence intensity of the apoenzyme.

	E·NADP^+^	E·Folate	E·NADP^+^·Folate
Wild-type	93%	36%	24%
midW22	102%	52%	49%
midW30	106%	42%	40%
midW47	92%	71%	54%
midW74	98%	64%	61%
midW133	93%	64%	63%

**Table 3:** The k_on_, k_off_, and K_d_ for the midW mutants determined by T-jump transient data analysis compared to the K_d_ determined by isothermal titration calorimetry (ITC).

Enzyme	k_on_ (μM^−1^s^−1^)	k_off_ (s^−1^)	K_d_ (μM) from T-jump	K_d_ (μM) from ITC
WT^a^	31 ± 2	1100 ± 200	35.5 ± 6.8	4.7
midW22	38 ± 20	3300 ± 1550	88 ± 61	3.7
midW30	51 ± 6	3400 ± 450	66 ± 12	4.5
midW47	22 ± 2	2000 ± 240	92 ± 14	12
midW74	53 ± 3	2900 ± 310	55.4 ± 6.6	8.4
midW133	51 ± 6	3100 ± 550	60 ± 13	9.5

^a^ Reported values from Reddish et al. [14].

**Table 4:** Average relaxation rates of the slow phase for the binary folate complexes of all five midW mutants and wt-DHFR.

Enzyme	midW22	midW30	midW47	midW74	midW133	wt-DHFR ^a^
Average Slow Rate (s^−1^)	2700 ± 900	1800 ± 960	740 ± 90	720 ± 180	370 ± 90	400 ± 70

^a^ Reported values from Reddish et al. [14].

## Supplementary Materials

The following are available online at https://www.mdpi.com/1420-3049/25/17/3819/s1, Additional information on the activity and melting temperatures of the midW mutants, as well as the ITC results and temperature dependence of the slow and fast relaxation rates are available online.
