# Supplementary material for: Site-Specific Tryptophan Labels Reveal Local Microsecond–Millisecond Motions of Dihydrofolate Reductase
Source: Molecules. 2020 Aug 22;25(17):3819. doi: 10.3390/molecules25173819 (PMC7503464; doi:10.3390/molecules25173819)
Supplement: Supplementary file 1 [file molecules-25-03819-s001.pdf]

**Supplemental Information**

Site-specific Tryptophan Labels Reveal Local Millisecond Motions of Dihydrofolate Reductase

Morgan B. Vaughn, Chloe Biren, Qun Li, Ashwin Ragupathi, R. Brian Dyer

**Table S1.** Relative activity of the midW mutants compared to wildtype DHFR

| **Enzyme** | wildtype | midW22 | midW30 | midW47 | midW74 | midW133 |
|---|---|---|---|---|---|---|
| **Relative Activity** | 100% | 90% | 33% | 20% | 20% | 22% |

*Isothermal Calorimetry*

The dissociation constant and thermodynamic parameters of folate binding to the midW mutants at 25°C were determined using isothermal calorimetry (ITC) on a MicroCal Auto-iTC200 from Malvern Panalytical (United Kingdom). The samples contained 350 µL of 100 µM enzyme and were injected with 2.4 µL of 1 mM folate (buffer: 50 mM sodium phosphate, 100 mM NaCl, pH 7). The system was allowed to equilibrate for 150 seconds between injections. Representative ITC data are shown in **Figure S1**. The ITC data were analyzed using the MicroCal ITC-ORIGIN Analysis Software. The resulting $K_d$, ΔH, and ΔS of ligand binding (**Table S2)** were used to calculate the sum of free concentrations, which were used to the determine the concentration dependence of the temperature jump relaxation rates.

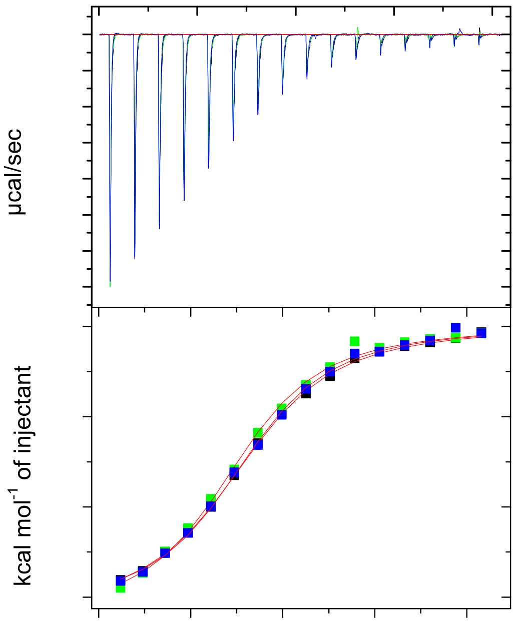


**Figure S1** Raw ITC data for folate binding to midW74 in triplicate (top). Processed ITC data for folate binding to midW74 in triplicate (bottom). The curves represent fits using the OneSite model in the MicroCal ITC-ORIGIN Analysis Software.

**Table S2** Dissociation and thermodynamic constants for the binding of folate to the five midW mutants.

| **midW mutant** | **$K_d$** (μM) | **ΔH** (kcal/mol) | **ΔS** (cal/mol/K) |
|---|---|---|---|
| midW22 | 3.7 | -7.78 | -1.26 |
| midW30 | 4.5 | -5.04 | 7.55 |
| midW47 | 12 | -7.03 | -1.16 |
| midW74 | 8.4 | -6.34 | 1.98 |
| midW133 | 9.4 | -5.70 | 3.85 |

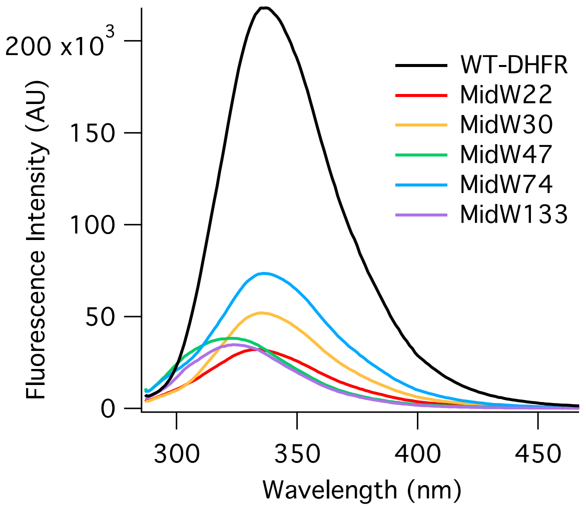


**Figure S2** Equilibrium fluorescence of the apoenzyme wt-DHFR and all of the midW mutants. To adjust for the different integration times, (0.5 s for wt-DHFR and 1.5 s for the midW mutants) the wt-DHFR spectra has been scaled by a factor of three.

*Intrinsic Temperature Dependence of Tryptophan Fluorescence*

The temperature dependent equilibrium fluorescence intensity of the midW mutants is corrected for the intrinsic change in tryptophan fluorescence with increasing temperature. The fluorescence spectra of free tryptophan were collected from 12ºC to 45ºC in increments of 3ºC using the same method described for the midW mutants. The fluorescence peak was integrated between 327 nm and 353 nm and normalized to the lowest temperature. The normalized fluorescence intensity vs temperature is shown in **Figure S3**. As the temperature increases, the fluorescence intensity of tryptophan decreases. The normalized fluorescence intensities of free tryptophan are then subtracted from the normalized fluorescence intensities of the midW mutants to correct for the effects of the intrinsic temperature dependence of tryptophan fluorescence.

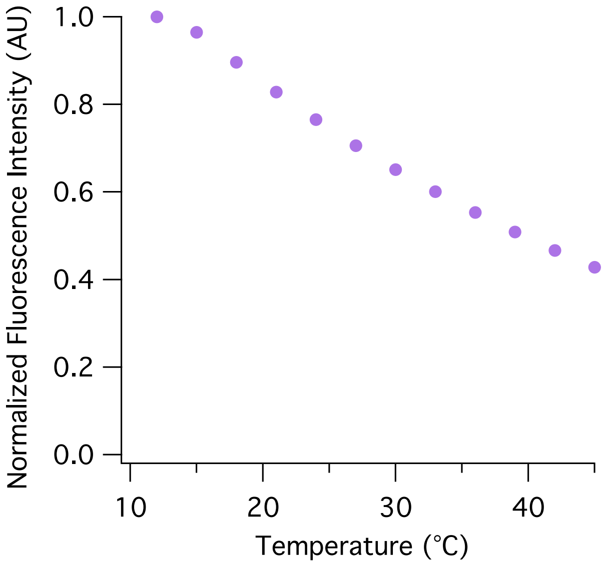


**Figure S3** Normalized fluorescence intensity of free tryptophan versus temperature.

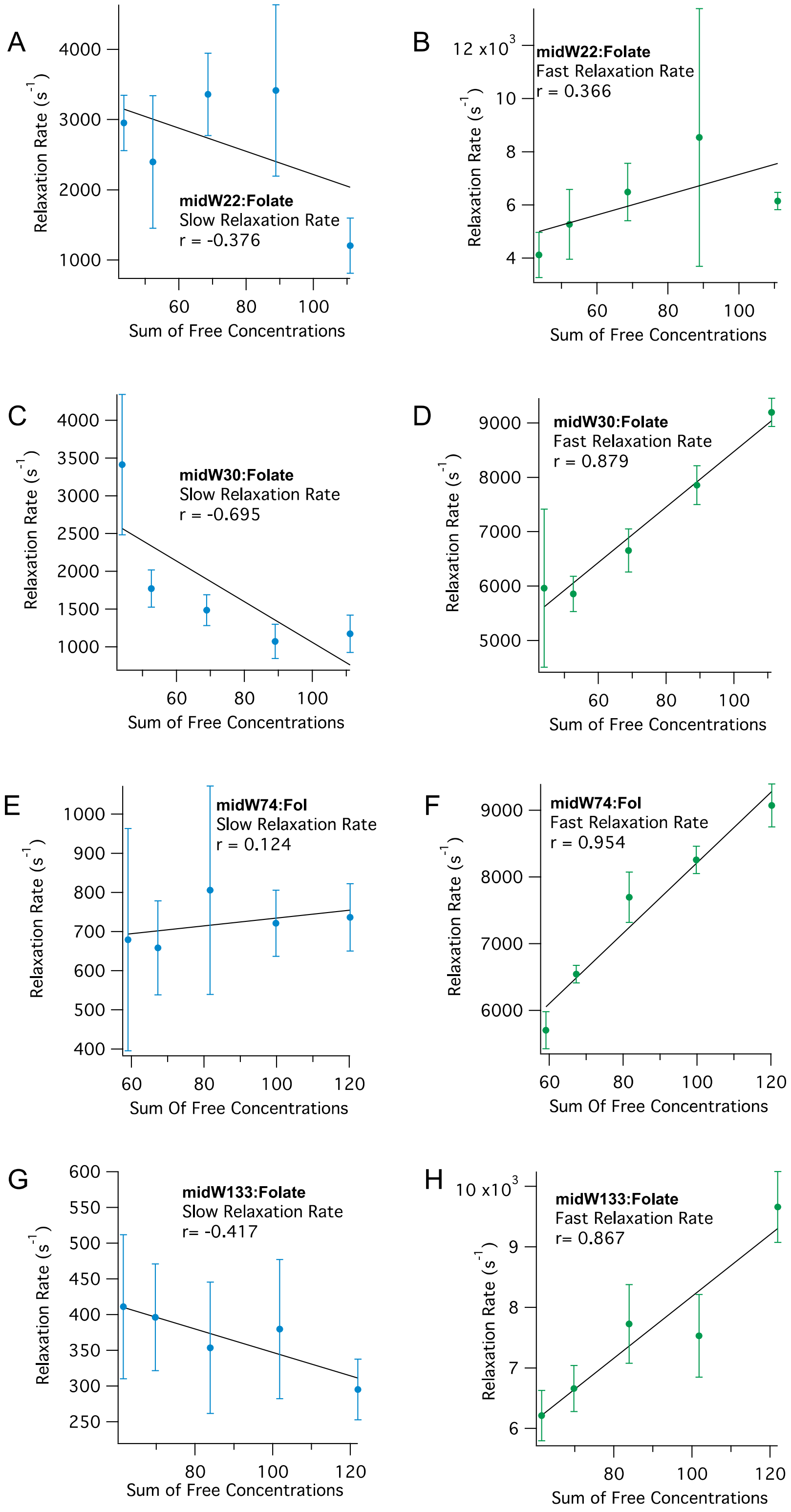


**Figure S4** Concentration dependence of the slow (A, C, E, G) and fast (B, D, F, G) relaxation rates for midW22, midW30, midW74, and midW133, respectively.

**Table S3** Average (n = 5) slow relaxation rates and amplitudes from double exponential fits of T-jump transients for each midW mutant sample

| **midW22** | Slow Relaxation Rate (s-1) | Slow Amplitude |
|---|---|---|
| 100 µM enzyme, 100 µM folate | 2950 ± 390 | 2.79 ± 0.64 |
| 100 µM enzyme, 125 µM folate | 2400 ± 950 | 5.5 ± 3.2 |
| 100 µM enzyme, 150 µM folate | 3360 ± 590 | 9.3 ± 3.9 |
| 100 µM enzyme, 175 µM folate | 3400 ± 1200 | 1.46 ± 0.86 |
| 100 µM enzyme, 200 µM folate | 1200 ± 390 | 1.63 ± 0.21 |
| | | |
| **midW30** | | |
| 100 µM enzyme, 100 µM folate | 3410 ± 930 | 4.5 ± 2.3 |
| 100 µM enzyme, 125 µM folate | 1770 ± 250 | 2.31 ± 0.48 |
| 100 µM enzyme, 150 µM folate | 1480 ± 200 | 2.28 ± 0.22 |
| 100 µM enzyme, 175 µM folate | 1070 ± 230 | 2.77 ± 0.18 |
| 100 µM enzyme, 200 µM folate | 1170 ± 250 | 2.354 ± 0.088 |
| | | |
| **midW47** | | |
| 100 µM enzyme, 100 µM folate | 760 ± 130 | 7.00 ± 0.69 |
| 100 µM enzyme, 125 µM folate | 767 ± 71 | 7.91 ± 0.38 |
| 100 µM enzyme, 150 µM folate | 690 ± 130 | 8.56 ± 1.4 |
| 100 µM enzyme, 175 µM folate | 746 ± 26 | 10.4 ± 1.0 |
| 100 µM enzyme, 200 µM folate | 754 ± 46 | 10.0 ± 2.0 |
| | | |
| **midW74** | | |
| 100 µM enzyme, 100 µM folate | 680 ± 280 | 2.15 ± 0.33 |
| 100 µM enzyme, 125 µM folate | 660 ± 120 | 2.14 ± 0.25 |
| 100 µM enzyme, 150 µM folate | 810 ± 270 | 2.73 ± 0.16 |
| 100 µM enzyme, 175 µM folate | 721 ± 85 | 3.07 ± 0.17 |
| 100 µM enzyme, 200 µM folate | 736 ± 86 | 3.65 ± 0.17 |
| | | |
| **midW133** | | |
| 100 µM enzyme, 100 µM folate | 410 ± 100 | 1.00 ± 0.19 |
| 100 µM enzyme, 125 µM folate | 396 ± 75 | 1.26 ± 0.26 |
| 100 µM enzyme, 150 µM folate | 353 ± 92 | 1.52 ± 0.36 |
| 100 µM enzyme, 175 µM folate | 380 ± 97 | 1.34 ± 0.37 |
| 100 µM enzyme, 200 µM folate | 295 ± 42 | 1.87 ± 0.34 |

**Table S4** Average (n = 5) fast relaxation rates and amplitudes from double exponential fits of T-jump transients for each midW mutant sample

| **midW22** | Fast Relaxation Rate (s-1) | Fast Amplitude |
|---|---|---|
| 100 µM enzyme, 100 µM folate | 4120 ± 850 | 1.89 ± 0.41 |
| 100 µM enzyme, 125 µM folate | 5300 ± 1200 | 5.4 ± 2.6 |
| 100 µM enzyme, 150 µM folate | 6500 ± 1100 | 5.5 ± 2.3 |
| 100 µM enzyme, 175 µM folate | 8500 ± 4800 | 1.4 ± 1.0 |
| 100 µM enzyme, 200 µM folate | 6150 ± 320 | 6.62 ± 0.79 |
| | | |
| **midW30** | | |
| 100 µM enzyme, 100 µM folate | 6000 ± 1500 | 4.7 ± 2.5 |
| 100 µM enzyme, 125 µM folate | 5860 ± 330 | 9.81 ± 0.79 |
| 100 µM enzyme, 150 µM folate | 6660 ± 400 | 9.75 ± 0.69 |
| 100 µM enzyme, 175 µM folate | 7860 ± 360 | 9.7 ± 1.0 |
| 100 µM enzyme, 200 µM folate | 9190 ± 260 | 8.2 ± 1.2 |
| | | |
| **midW47** | | |
| 100 µM enzyme, 100 µM folate | 3430 ± 280 | 8.15 ± 0.64 |
| 100 µM enzyme, 125 µM folate | 3820 ± 170 | 7.96 ± 1.1 |
| 100 µM enzyme, 150 µM folate | 3850 ± 160 | 8.3 ± 1.9 |
| 100 µM enzyme, 175 µM folate | 4630 ± 190 | 7.9 ± 0.7 |
| 100 µM enzyme, 200 µM folate | 4640 ± 190 | 8.1 ± 1.5 |
| | | |
| **midW74** | | |
| 100 µM enzyme, 100 µM folate | 5700 ± 280 | 10.77 ± 0.90 |
| 100 µM enzyme, 125 µM folate | 6540 ± 130 | 10.37 ± 0.68 |
| 100 µM enzyme, 150 µM folate | 7700 ± 380 | 10.83 ± 0.99 |
| 100 µM enzyme, 175 µM folate | 8260 ± 200 | 10.73 ± 0.79 |
| 100 µM enzyme, 200 µM folate | 9070 ± 320 | 10.83 ± 0.48 |
| | | |
| **midW133** | | |
| 100 µM enzyme, 100 µM folate | 6220 ± 420 | 3.17 ± 0.26 |
| 100 µM enzyme, 125 µM folate | 6660 ± 380 | 3.80 ± 0.18 |
| 100 µM enzyme, 150 µM folate | 7730 ± 650 | 3.11 ± 0.14 |
| 100 µM enzyme, 175 µM folate | 7530 ± 680 | 2.72 ± 0.22 |
| 100 µM enzyme, 200 µM folate | 9660 ± 580 | 2.96 ± 0.19 |
